# Supplementary material for: Net Conversion of Human-Edible Vitamins and Minerals in the U.S. Southern Great Plains Beef Production System
Source: Animals (Basel). 2022 Aug 24;12(17):2170. doi: 10.3390/ani12172170 (PMC9454978; doi:10.3390/ani12172170)
Supplement: Supplementary file 1 [file animals-12-02170-s001.zip › animals-1809903-supplementary.pdf]

SUPPLEMENTARY MATERIAL

Table S1. Beef product yield (% shrunk body weight) estimates used to compute human-edible nutrient production

Animal	Meat	Liver	Heart	Kidney	Spleen	Pancreas	GIT[1]
Mature cow	32.98	1.0	0.34	0.17	0.11	0.07	3.15
Bull	29.54	1.0	0.34	0.17	0.11	0.07	3.15
Weaned steer	36.51	1.2	0.45	0.26	0.19	0.08	5.85
Weaned heifer	36.51	1.4	0.45	0.26	0.19	0.08	5.85
Heifer at breeding	38.23	1.4	0.45	0.27	0.21	0.11	5.57
Steer entering feedlot	38.23	1.4	0.45	0.27	0.21	0.11	5.57
Heifer entering feedlot	38.23	1.4	0.45	0.27	0.21	0.11	5.57
Finished steer	41.39	1.4	0.45	0.21	0.19	0.09	5.15
Finished heifer	41.39	1.4	0.45	0.21	0.19	0.09	5.15

[1] GIT = Gastrointestinal tract

Table S2. Nutrient concentration of feedstuffs, red meat, and organ meats used in summative model

Item[1]	Iron, ppm	Zinc, ppm	Selenium, ppm	Phosphorus, %	B6, ppm	Riboflavin, ppm	Niacin, ppm	Choline, ppm	Tryptophan, ppm
Alfalfa hay	240	23.5	0.55	0.26	4.4	13	42	1,200	567
Bermudagrass	236	37.5	0.24	0.25	NA	10	47	744	526
Cottonseed meal	150	63.9	1.29	1.13	5.8	4.9	44	3,076	5,300
Corn grain	50	20.5	0.61	0.29	7.6	1.2	27	702	600
Corn silage	50	20.5	0.61	0.29	7.6	1.2	27	702	600
Distiller's grains	103	63.3	0.99	0.86	10	8.6	82	2,963	2,100
Mineral, cow-calf	2,500	3,600	18.25	5.6	0	0	0	0	0
Mineral, stocker	2,500	2,850	21.5	4.5	0	0	0	0	0
Mineral, feedlot	800	3,500	8	3.6	0	0	0	0	0
Molasses	263	17.4	5.33	0.22	9.1	4.0	10	180	10
Tallow	482	42	0	NA	0	0	0	80	0
Red meat	26	59.1	0.27	21.08	4.2	2.24	51	909	2,580
Liver	63	52.3	0.34	48.7	10.1	34	173.4	4,186	2,800
Heart	46	19	0.21	21.1	2.42	8.65	63	1,700	2,400
Kidney	75	24	2.73	22.9	4.45	23	66	3,000	2,530
Spleen	504	26	1.14	25.2	1.2	0.7	48	4,000	2,300
Pancreas	45	44	NA[2]	30.4	2.0	3.1	39	NA	NA
Gastrointestinal tract	31	15.5	NA	15.1	NA	0.59	19	NA	NA

[1]Nutrient concentrations are on dry matter basis for feedstuffs and wet basis for red and organ meats.
[2]NA = no data on nutrient concentration were available

LITERATURE USED TO DETERMINE NUTRIENT CONCENTRATIONS

Abdilova, G., M. Rebezov, A. Nesterenko, S. Safronov, I. Knysh, I. Ivanova, I. Mikolaychik, and L. Morozova. 2021. Characteristics of meat by-products: nutritional and biological value. International Journal of Modern Agriculture. 10:3895–3904.

Adams, J. F., F. Mcewan, and A. Wilson. 1973. The vitamin B12 content of meals and items of diet. British Journal of Nutrition. 29:65–72. doi:10.1079/BJN19730078.

Arthur, D. 1972. Selenium Content of Canadian Foods. Canadian Institute of Food Science and Technology Journal. 5:165–169. doi:10.1016/S0315-5463(72)74114-0.

Biel, W., E. Czerniawska-Piątkowska, and A. Kowalczyk. 2019. Offal Chemical Composition from Veal, Beef, and Lamb Maintained in Organic Production Systems. Animals. 9:489. doi:10.3390/ani9080489.

Daun, C., and B. Åkesson. 2004. Glutathione peroxidase activity, and content of total and soluble selenium in five bovine and porcine organs used in meat production. Meat Science. 66:801–807. doi:10.1016/S0309-1740(03)00178-5.

Elvehjem, C. A., and W. H. Peterson. 1927. The iron content of animal tissues. Journal of Biological Chemistry. 74:433–441. doi:10.1016/S0021-9258(20)74035-4.

Engel, R. W. 1943. The Choline Content of Animal and Plant Products. The Journal of Nutrition. 25:441–446. doi:10.1093/jn/25.5.441.

Forbes, E. B., and R. W. Swift. 1926. The iron content of meats. Journal of Biological Chemistry. 67:517–521. doi:10.1016/S0021-9258(18)84721-4.

Francis, C. K., and P. F. Trowbridge. 1910. Phosphorus in Beef Animals. Part II. Journal of Biological Chemistry. 8:81–93. doi:10.1016/S0021-9258(18)91544-9.

Gille, D., and A. Schmid. 2015. Vitamin B12 in meat and dairy products. Nutrition Reviews. 73:106–115. doi:10.1093/nutrit/nuu011.

van Heerden, S. M., and L. Morey. 2014. Nutrient content of South African C2 beef offal. Food Measure. 8:249–258. doi:10.1007/s11694-014-9198-z.

Henderson, L. M., H. A. Waisman, and C. A. Elvehjem. 1941. The distribution of pyridoxine (vitamin B6) in meat and meat products. Journal of Nutrition. 21:589–598.

Howe, J.C.; Williams, J.; Holden, J.M.; Zeisel, S.H.; Mar, M.-H. USDA Database for the Choline Content of Common Foods Available online: http://www.nal.usda.gov/fnic/foodcomp/Data/Choline/ Choline.pdf (accessed on 5 June 2021).

Kesterson, H. F. 2017. Nutrient analysis of ten raw U.S. beef variety meat items and beef flavor myology [Text]. Colorado State University. Available from: https://mountainscholar.org/handle/10217/191483

Kizlaitis, L., M. I. Steinfeld, and A. J. Siedler. 1962. Nutrient Content of Variety Meats. Journal of Food Science. 27:459–462. doi:10.1111/j.1365-2621.1962.tb00127.x.

Lawler, T. L., J. B. Taylor, J. W. Finley, and J. S. Caton. 2004. Effect of supranutritional and organically bound selenium on performance, carcass characteristics, and selenium distribution in finishing beef steers1. Journal of Animal Science. 82:1488–1493. doi:10.2527/2004.8251488x.

Li, C. 2017. The role of beef in human nutrition and health. In: Ensuring Safety and Quality in the Production of Beef p. 329–338. Cambridge, UK: Burleigh Dodds Science Publishing, https://doi.org/10.19103/AS.2016.0009.16.

Luecke, R. W., and P. B. Pearson. 1944. The determination of free choline in animal tissues. Journal of Biological Chemistry. 155:507–512. doi:10.1016/S0021-9258(18)51181-9.

McIntire, J. M., B. S. Schweigert, and C. A. Elvehjem. 1944. The Choline and Pyridoxine Content of Meats. The Journal of Nutrition. 28:219–223. doi:10.1093/jn/28.4.219.

Morris, V. C., and O. A. Levander. 1970. Selenium Content of Foods. The Journal of Nutrition. 100:1383–1388. doi:10.1093/jn/100.12.1383.

National Academies of Sciences, Engineering, and Medicine. 2016. Nutrient Requirements of Beef Cattle: Eighth Revised Edition. The National Academies Press, Washington, DC. Available from: https://www.nap.edu/catalog/19014/nutrient-requirements-of-beef-cattle-eighth-revised-edition

National Research Council. 1982. United States-Canadian Tables of Feed Composition: Nutritional Data for United States and Canadian Feeds, Third Revision. 3rd ed. National Academies Press, Washington, D.C.

National Research Council. 1994. Nutrient Requirements of Poultry: Ninth Revised Edition. 9th ed. National Academies Press, Washington, D.C.

National Research Council. 2012. Nutrient Requirements of Swine: Eleventh Revised Edition. 11th ed. National Academy Press, Washington, DC.

Ockerman, H. W., and L. Basu. 2004. By-products. In: C. Devine and M. Dikeman, editors. Encyclopedia of Meat Sciences. Elsevier Academic Press Inc, Amsterdam, London. p. 104–112.

Rouser, G., G. Simon, and G. Kritchevsky. 1969. Species variations in phospholipid class distribution of organs: I. Kidney, liver and spleen. Lipids. 4:599–606. doi:10.1007/BF02531047.

Scheid, H. E., M. M. Andrews, and B. S. Schweigert. 1952. Comparison of Methods for Determination of the Vitamin B12 Potency of Meats: One Figure. The Journal of Nutrition. 47:601–610. doi:10.1093/jn/47.4.601.

Scheid, H. E., and B. S. Schweigert. 1954. Vitamin B12 Content of Organ Meats. The Journal of Nutrition. 53:419–427. doi:10.1093/jn/53.3.419.

Seong, P. N., G. H. Kang, K. M. Park, S. H. Cho, S. M. Kang, B. Y. Park, S. S. Moon, and H. V. Ba. 2014. Characterization of Hanwoo Bovine By-products by Means of Yield, Physicochemical and Nutritional Compositions. Korean J Food Sci Anim Resour. 34:434–447. doi:10.5851/kosfa.2014.34.4.434.

Simon, G., and G. Rouser. 1969. Species variations in phospholipid class distribution of organs: II. Heart and skeletal muscle. Lipids. 4:607–614. doi:10.1007/BF02531048.

Ullrey, D. E., P. S. Brady, P. A. Whetter, P. K. Ku, and W. T. Magee. 1977. Selenium Supplementation of Diets for Sheep and Beef Cattle. Journal of Animal Science. 45:559–565. doi:10.2527/jas1977.453559x.

United States Department of Agriculture. 2018. Food and Nutrient Database for Dietary Studies. Available from: https://data.nal.usda.gov/

Valenzuela, C., D. López de Romaña, M. Olivares, M. S. Morales, and F. Pizarro. 2009. Total Iron and Heme Iron Content and their Distribution in Beef Meat and Viscera. Biol Trace Elem Res. 132:103–111. doi:10.1007/s12011-009-8400-3.

Williams, P. 2007. Nutritional composition of red meat. Nutrition & Dietetics. 64:S113–S119. doi:10.1111/j.1747-0080.2007.00197.x.

Table S3. Nutrient absorption coefficients (%) of feedstuffs, red meat, and organ meats used in summative model.

Item	Iron	Zinc	Selenium	Phosphorus	B12	B6	Riboflavin	Niacin	Choline	Tryptophan
Alfalfa hay	0	0	0	0	0	0	0	0	0	0
Bermudagrass	0	0	0	0	0	0	0	0	0	0
Cottonseed meal	0	0	0	0	0	0	0	0	0	0
Corn grain	6	21.75	80	28	0	53.5	63	40	75	80
Corn silage	6	21.75	80	28	0	53.5	63	40	75	80
Distiller's grains	0	0	0	0	0	0	0	0	0	0
Mineral, cow-calf	0	11.2	54	85	0	0	0	0	0	0
Mineral, stocker	0	11.2	54	85	0	0	0	0	0	0
Mineral, feedlot	0	11.2	54	85	0	0	0	0	0	0
Molasses	5	15	86	63	0	75	63	100	56	86
Tallow	0	0	0	0	0	0	0	0	0	0
Red meat	18.13	36.34	89	69	66.25	89	89	84.5	95	97.5
Organ meats	11	14.3	89	65.5	23.57	89	95	100	88	96

LITERATURE USED TO DETERMINE NUTRIENT ABSORPTION COEFFICIENTS

Adams, C. L., M. Hambidge, V. Raboy, J. A. Dorsch, L. Sian, J. L. Westcott, and N. F. Krebs. 2002. Zinc absorption from a low–phytic acid maize. The American Journal of Clinical Nutrition. 76:556–559. doi:10.1093/ajcn/76.3.556.

Almeida, F. N., G. I. Petersen, and H. H. Stein. 2011. Digestibility of amino acids in corn, corn coproducts, and bakery meal fed to growing pigs. Journal of Animal Science. 89:4109–4115. doi:10.2527/jas.2011-4143.

Aoyagi, S., K. M. Hiney, and D. H. Baker. 1995. Estimates of zinc and iron bioavailability in pork liver and the effect of sex of pig on the bioavailability of copper in pork liver fed to male and female chicks. Journal of Animal Science. 73:793–798. doi:10.2527/1995.733793x.

Ball, G. F. M. 1998. Niacin and tryptophan. In: G. F. M. Ball, editor. Bioavailability and Analysis of Vitamins in Foods. Springer US, Boston, MA. p. 319–359. Available from: https://doi.org/10.1007/978-1-4899-3414-7_9

Ball, G. F. M. 1998. Riboflavin and other flavins (vitamin B2). In: G. F. M. Ball, editor. Bioavailability and Analysis of Vitamins in Foods. Springer US, Boston, MA. p. 319–359. Available from: https://doi.org/10.1007/978-1-4899-3414-7_9

Bindari, Y. R., H. N. Lærke, and J. V. Nørgaard. 2018. Standardized ileal digestibility and digestible indispensable amino acid score of porcine and bovine hydrolyzates in pigs. Journal of the Science of Food and Agriculture. 98:2131–2137. doi:10.1002/jsfa.8697.

Bohlke, R. A., R. C. Thaler, and H. H. Stein. 2005. Calcium, phosphorus, and amino acid digestibility in low-phytate corn, normal corn, and soybean meal by growing pigs1,2. Journal of Animal Science. 83:2396–2403. doi:10.2527/2005.83102396x.

Brnić, M., R. Wegmüller, C. Zeder, G. Senti, and R. F. Hurrell. 2014. Influence of Phytase, EDTA, and Polyphenols on Zinc Absorption in Adults from Porridges Fortified with Zinc Sulfate or Zinc Oxide. The Journal of Nutrition. 144:1467–1473. doi:10.3945/jn.113.185322.

Budowski, P., I. Kafri, and D. Sklan. 1977. Utilization of Choline From Crude Soybean Lecithin by Chicks: 2. Absorption Measurements. Poultry Science. 56:754–757. doi:10.3382/ps.0560754.

Carter, E. G. A., and K. J. Carpenter. 1982. The Available Niacin Values of Foods for Rats and Their Relation to Analytical Values. The Journal of Nutrition. 112:2091–2103. doi:10.1093/jn/112.11.2091.

Çatak, J. 2019. Determination of niacin profiles in some animal and plant based foods by high performance liquid chromatography: association with healthy nutrition. J Anim Sci Technol. 61:138–146. doi:10.5187/jast.2019.61.3.138.

Cervantes-Pahm, S. K., Y. Liu, and H. H. Stein. 2014. Digestible indispensable amino acid score and digestible amino acids in eight cereal grains. British Journal of Nutrition. 111:1663–1672. doi:10.1017/S0007114513004273.

Chomba, E., C. M. Westcott, J. E. Westcott, E. M. Mpabalwani, N. F. Krebs, Z. W. Patinkin, N. Palacios, and K. M. Hambidge. 2015. Zinc Absorption from Biofortified Maize Meets the Requirements of Young Rural Zambian Children. The Journal of Nutrition. 145:514–519. doi:10.3945/jn.114.204933.

Christensen, M. J., M. Janghorbani, F. H. Steinke, N. Istfan, and V. R. Young. 1983. Simultaneous determination of absorption of selenium from poultry meat and selenite in young men: application of a triple stable-isotope method. British Journal of Nutrition. 50:43–50. doi:10.1079/BJN19830070.

Chung, T. K., and D. H. Baker. 1990. Riboflavin Requirement of Chicks Fed Purified Amino Acid and Conventional Corn-Soybean Meal Diets. Poultry Science. 69:1357–1363. doi:10.3382/ps.0691357.

Cook, J. D., M. B. Reddy, J. Burri, M. A. Juillerat, and R. F. Hurrell. 1997. The influence of different cereal grains on iron absorption from infant cereal foods. The American Journal of Clinical Nutrition. 65:964–969. doi:10.1093/ajcn/65.4.964.

Emmert, J. L., and D. H. Baker. 1997. A Chick Bioassay Approach for Determining the Bioavailable Choline Concentration in Normal and Overheated Soybean Meal, Canola Meal and Peanut Meal. The Journal of Nutrition. 127:745–752. doi:10.1093/jn/127.5.745.

Farouk, M. M., G. Wu, D. A. Frost, M. Staincliffe, and S. O. Knowles. 2019. Factors Affecting the Digestibility of Beef and Consequences for Designing Meat-Centric Meals. Journal of Food Quality. 2019:e2590182. doi:10.1155/2019/2590182.

Finley, J. W. 1999. The Retention and Distribution by Healthy Young Men of Stable Isotopes of Selenium Consumed as Selenite, Selenate or Hydroponically-Grown Broccoli Are Dependent on the Isotopic Form. The Journal of Nutrition. 129:865–871. doi:10.1093/jn/129.4.865.

Gallaher, D. D., P. E. Johnson, J. R. Hunt, G. I. Lykken, and M. J. Marchello. 1988. Bioavailability in humans of zinc from beef: intrinsic vs extrinsic labels. The American Journal of Clinical Nutrition. 48:350–354. doi:10.1093/ajcn/48.2.350.

Gregory, J. F., III, P. R. Trumbo, L. B. Bailey, J. P. Toth, T. G. Baumgartner, and J. J. Cerda. 1991. Bioavailability of Pyridoxine-5′-β-D-Glucoside Determined in Humans by Stable-Isotopic Methods. The Journal of Nutrition. 121:177–186. doi:10.1093/jn/121.2.177.

Griffiths, N. M., R. D. H. Stewart, and M. F. Robinson. 1976. The metabolism of [75Se] selenomethionine in four women. British Journal of Nutrition. 35:373–382. doi:10.1079/BJN19760043.

Hambidge, K. M., J. W. Huffer, V. Raboy, G. K. Grunwald, J. L. Westcott, L. Sian, L. V. Miller, J. A. Dorsch, and N. F. Krebs. 2004. Zinc absorption from low-phytate hybrids of maize and their wild-type isohybrids. The American Journal of Clinical Nutrition. 79:1053–1059. doi:10.1093/ajcn/79.6.1053.

Harris, R. S., L. M. Mosher, and J. W. M. Bunker. 1939. The nutritional availability of iron in molasses. American Journal of Digestive Diseases. 6:459–462.

Heyssel, R. M., R. C. Bozian, W. J. Darby, and M. C. Bell. 1966. Vitamin B12 turnover in man. The assimilation of vitamin B12 from natural foodstuff by man and estimates of minimal daily dietary requirements. American Journal of Clinical Nutrition. 18:176–184.

Hodgkinson, S. M., C. A. Montoya, P. T. Scholten, S. M. Rutherfurd, and P. J. Moughan. 2018. Cooking Conditions Affect the True Ileal Digestible Amino Acid Content and Digestible Indispensable Amino Acid Score (DIAAS) of Bovine Meat as Determined in Pigs. The Journal of Nutrition. 148:1564–1569. doi:10.1093/jn/nxy153.

Institute of Medicine. 1998. Dietary Reference Intakes for Thiamin, Riboflavin, Niacin, Vitamin B6, Folate, Vitamin B12, Pantothenic Acid, Biotin, and Choline. The National Academies Press, Washington, DC. Available from: https://www.nap.edu/catalog/6015/dietary-reference-intakes-for-thiamin-riboflavin-niacin-vitamin-b6-folate-vitamin-b12-pantothenic-acid-biotin-and-choline

Institute of Medicine. 2000. Dietary Reference Intakes for Vitamin C, Vitamin E, Selenium, and Carotenoids. The National Academies Press, Washington, DC. Available from: https://www.nap.edu/catalog/9810/dietary-reference-intakes-for-vitamin-c-vitamin-e-selenium-and-carotenoids

Itkonen, S. T., H. J. Karp, and C. J. E. Lamberg-Allardt. 2017. Bioavailability of Phosphorus. In: J. Uribarri and M. S. Calvo, editors. Dietary Phosphorus: Health, nutrition, and regulatory aspects. p. 221–233.

Johnson, J. M., and P. M. Walker. 1992. Zinc and iron utilization in young women consuming a beef-based diet. Journal of the American Dietetic Association. 92:1474–1478. doi:10.1016/S0002-8223(21)00930-5.

Johnson, P. E., D. D. Gallaher, G. I. Lykken, and J. R. Hunt. 1990. Zinc availability from beef served with various carbohydrates or beverages. Nutrition Research. 10:155–162. doi:10.1016/S0271-5317(05)80603-7.

Layrisse, M., J. D. Cook, C. Martinez, M. Roche, I. N. Kuhn, R. B. Walker, and C. A. Finch. 1969. Food Iron Absorption: A Comparison of Vegetable and Animal Foods. Blood. 33:430–443. doi:10.1182/blood.V33.3.430.430.

Li, S. F., Y. B. Niu, J. S. Liu, L. Lu, L. Y. Zhang, C. Y. Ran, M. S. Feng, B. Du, J. L. Deng, and X. G. Luo. 2013. Energy, amino acid, and phosphorus digestibility of phytase transgenic corn for growing pigs. Journal of Animal Science. 91:298–308. doi:10.2527/jas.2012-5211.

Long, Z., and M. S. Pittman. 1935. Utilization of Meat by Human Subjects: II. The Utilization of the Nitrogen and Phosphorus of Round and Liver of Beef. The Journal of Nutrition. 9:677–683. doi:10.1093/jn/9.6.677.

Martínez-Torres, C., and M. Layrisse. 1971. Iron absorption from veal muscle. The American Journal of Clinical Nutrition. 24:531–540. doi:10.1093/ajcn/24.5.531.

Maseta, E., T. C. Mosha, C. Nyaruhucha, and H. Laswai. 2017. Nutritional quality of quality protein maize-based supplementary foods. Nutrition & Food Science. 47:42–52. doi:10.1108/NFS-04-2016-0042.

Mazariegos, M., K. M. Hambidge, N. F. Krebs, J. E. Westcott, S. Lei, G. K. Grunwald, R. Campos, B. Barahona, V. Raboy, and N. W. Solomons. 2006. Zinc absorption in Guatemalan schoolchildren fed normal or low-phytate maize. The American Journal of Clinical Nutrition. 83:59–64. doi:10.1093/ajcn/83.1.59.

McClellan, W. S., V. R. Rupp, and V. Toscani. 1930. Clinical Calorimetry. XLVI. Prolonged meat diets with a study of the metabolism of nitrogen, calcium, and phosphorus. Journal of Biological Chemistry. 87:669–680. doi:10.1016/S0021-9258(18)76843-9.

Mendoza, C., F. E. Viteri, B. Lönnerdal, K. A. Young, V. Raboy, and K. H. Brown. 1998. Effect of genetically modified, low-phytic acid maize on absorption of iron from tortillas. The American Journal of Clinical Nutrition. 68:1123–1127. doi:10.1093/ajcn/68.5.1123.

Menten, J., G. Pesti, and R. Bakalli. 1997. A new method for determining the availability of choline in soybean meal. Poultry Science. 76:1292–1297. doi:10.1093/ps/76.9.1292.

Moser-Veillon, P., A. Reed Mangels, K. Y. Patterson, and C. Veillon. 1992. Utilization of two different chemical forms of selenium during lactation using stable isotope tracers: an example of speciation in nutrition. Analyst. 117:559–562. doi:10.1039/AN9921700559.

Nakano, H., L. G. McMahon, and J. F. Gregory III. 1997. Pyridoxine-5′-β-D-glucoside Exhibits Incomplete Bioavailability as a Source of Vitamin B-6 and Partially Inhibits the Utilization of Co-Ingested Pyridoxine in Humans. The Journal of Nutrition. 127:1508–1513. doi:10.1093/jn/127.8.1508.

National Research Council. 2012. Nutrient Requirements of Swine: Eleventh Revised Edition. 11th ed. National Academy Press, Washington, DC.

Nyberg, W., and P. Reizenstein. 1958. Intestinal absorption of radiovitamin B12 bound in pig liver. Lancet. 2:832–833.

Oberli, M., A. Marsset-Baglieri, G. Airinei, V. Santé-Lhoutellier, N. Khodorova, D. Rémond, A. Foucault-Simonin, J. Piedcoq, D. Tomé, G. Fromentin, R. Benamouzig, and C. Gaudichon. 2015. High True Ileal Digestibility but Not Postprandial Utilization of Nitrogen from Bovine Meat Protein in Humans Is Moderately Decreased by High-Temperature, Long-Duration Cooking. The Journal of Nutrition. 145:2221–2228. doi:10.3945/jn.115.216838.

O'Dell, B. L., and R. A. Sunde, eds. 1997. Handbook of Nutritionally Essential Mineral Elements. Marcel Dekker, New York, NY.

O'Leary, F., and S. Samman. 2010. Vitamin B12 in Health and Disease. Nutrients. 2:299–316. doi:10.3390/nu2030299.

Patterson, B. H., O. A. Levander, K. Helzlsouer, P. A. McAdam, S. A. Lewis, P. R. Taylor, C. Veillon, and L. A. Zech. 1989. Human selenite metabolism: a kinetic model. American Journal of Physiology-Regulatory, Integrative and Comparative Physiology. 257:R556–R567. doi:10.1152/ajpregu.1989.257.3.R556.

Pittman, M. S., and B. L. Kunerth. 1939. A longtime study of nitrogen, calcium and phosphorus metabolism on a medium-protein diet. Journal of Nutrition. 17:175–185.

Reizenstein, P. G., and W. Nyberg. 1959. Intestinal absorption of liver-bound radiovitamin B12 in patients with pernicious anaemia and in controls. Lancet. 2:248–252.

Rohse, W. G., and G. W. Searle. 1955. Absorption of Choline From Intestinal Loops in Dogs. American Journal of Physiology-Legacy Content. 181:207–209. doi:10.1152/ajplegacy.1955.181.1.207.

de Romaña, D. L., B. Lönnerdal, and K. H. Brown. 2003. Absorption of zinc from wheat products fortified with iron and either zinc sulfate or zinc oxide. The American Journal of Clinical Nutrition. 78:279–283. doi:10.1093/ajcn/78.2.279.

Roth-Maier, D. A., S. I. Kettler, and M. Kirchgessner. 2002. Availability of vitamin B 6 from different food sources. International Journal of Food Sciences and Nutrition. 53:171–179. doi:10.1080/09637480220132184.

Roth-Maier, D. A., and M. Kirchgessner. 1996. [Investigations on the precaecal digestibility of natural thiamine, riboflavin and natural pantothenic acid in the swine animal model]. Z Ernahrungswiss. 35:318–322. doi:10.1007/bf01610549.

Roth-Maier, D. A., M. Kirchgessner, W. Erhardt, J. Henke, and U. Hennig. 1998. Comparative studies for the determination of precaecal digestibility as a measure for the availability of B-vitamins. Journal of Animal Physiology and Animal Nutrition. 79:198–209. doi:10.1111/j.1439-0396.1998.tb00643.x.

Roth-Maier, D., A. Wauer, G. Stangl, and M. Kirchgessner. 2000. Precaecal Digestibility of Niacin and Pantothenic Acid from Different Foods. International Journal for Vitamin and Nutrition Research. 70:8–13. doi:10.1024/0300-9831.70.1.8.

Schuette, S. A., and H. M. Linkswiler. 1982. Effects on Ca and P Metabolism in Humans by Adding Meat, Meat Plus Milk, or Purified Proteins Plus Ca and P to a Low Protein Diet. The Journal of Nutrition. 112:338–349. doi:10.1093/jn/112.2.338.

Sirichakwal, P. P., V. R. Young, and M. Janghorbani. 1985. Absorption and retention of selenium from intrinsically labeled egg and selenite as determined by stable isotope studies in humans. The American Journal of Clinical Nutrition. 41:264–269. doi:10.1093/ajcn/41.2.264.

Spencer, J. D., G. L. Allee, and T. E. Sauber. 2000. Phosphorus bioavailability and digestibility of normal and genetically modified low-phytate corn for pigs. Journal of Animal Science. 78:675–681. doi:10.2527/2000.783675x.

Thomson, C. D., and M. F. Robinson. 1986. Urinary and fecal excretions and absorption of a large supplement of selenium: superiority of selenate over selenite. The American Journal of Clinical Nutrition. 44:659–663. doi:10.1093/ajcn/44.5.659.

Tran, C. D., L. V. Miller, N. F. Krebs, S. Lei, and K. M. Hambidge. 2004. Zinc absorption as a function of the dose of zinc sulfate in aqueous solution. The American Journal of Clinical Nutrition. 80:1570–1573. doi:10.1093/ajcn/80.6.1570.

Tsubaki, H., and T. Komai. 1987. Intestinal absorption of choline in rats. J Pharmacobio-Dyn. 10:571–579.

Turnbull, A., F. Cleton, and C. A. Finch. 1962. Iron absorption. IV. The absorption of hemoglobin iron. J Clin Invest. 41:1897–1907.

Vrzhesinskaia, O A, V M Kodentsova, and V B Spirichev. "Absorption of vitamin B2 from plant and animal food products." *Fiziolohichnyi zhurnal (Kiev, Ukraine* 40, no. 1 (January 1, 1994): 39–47.

Wauer, A., G. I. Stangl, M. Kirchgessner, W. Erhardt, J. Henke, U. Hennig, and D. A. Roth-Maier. 1999. A comparative evaluation of ileo-rectal anastomosis techniques for the measurement of apparent precaecal digestibilities of folate, niacin and pantothenic acid. Journal of Animal Physiology and Animal Nutrition. 82:80–87. doi:10.1111/j.1439-0396.1999.00227.x.

Weremko, D., H. Fandrejewski, T. Zebrowska, I. K. Han, J. H. Kim, and W. T. Cho. 1997. Bioavailability of Phosphorus in Feeds of Plant Origin for Pigs - Review -. Asian-Australasian Journal of Animal Sciences. 10:551–566. doi:10.5713/ajas.1997.551.

Yen, J. T., A. H. Jensen, and D. H. Baker. 1976. Assessment of the Concentration of Biologically Available Vitamin B-6 in Corn and Soybean Meal. Journal of Animal Science. 42:866–870. doi:10.2527/jas1976.424866x.

Young, V. R., and P. L. Pellett. 1994. Plant proteins in relation to human protein and amino acid nutrition. The American Journal of Clinical Nutrition. 59:1203S-1212S. doi:10.1093/ajcn/59.5.1203S.

Zhai, H., and O. Adeola. 2013. True total-tract digestibility of phosphorus in corn and soybean meal for fifteen-kilogram pigs are additive in corn–soybean meal diet. Journal of Animal Science. 91:219–224. doi:10.2527/jas.2012-5295.

Zheng, J. J., J. B. Mason, I. H. Rosenberg, and R. J. Wood. 1993. Measurement of zinc bioavailability from beef and a ready-to-eat high-fiber breakfast cereal in humans: application of a whole-gut lavage technique. The American Journal of Clinical Nutrition. 58:902–907. doi:10.1093/ajcn/58.6.902.

Zierenberg, O., and S. M. Grundy. 1982. Intestinal absorption of polyenephosphatidylcholine in man. Journal of Lipid Research. 23:1136–1142. doi:10.1016/S0022-2275(20)38050-0.
